# Atomistic Insights into Graphene Oxide Dot Interactions with Integrin α_V_β_3_ from Microsecond Simulations

**DOI:** 10.3390/nano16140896

**Published:** 2026-07-22

**Authors:** Giulia Frigerio, Jules Grollier, Paulo Siani, Edoardo Donadoni, Cristiana Di Valentin

**Affiliations:** 1 Department of Materials Science, University of Milano-Bicocca, Via R. Cozzi 55, 20125 Milan, Italy; jules.grollier@uni-saarland.de (J.G.); paulo.siani@unimib.it (P.S.); edoardo.donadoni@unimib.it (E.D.); 2 BioNanoMedicine Center NANOMIB, University of Milano-Bicocca, 20125 Milan, Italy

**Keywords:** active targeting, nanoparticle, cancer therapy, ligand, RGD, molecular dynamics, polyethylene glycol, nanomedicine, protein receptor, inorganic nanomaterial

## Abstract

Graphene oxide (GO)-based nanomaterials functionalized with targeting ligands are promising platforms for selective cancer drug delivery. Among relevant targets, integrin α_V_β_3_ is a highly overexpressed receptor in several solid tumors and is commonly targeted using cyclic Arg-Gly-Asp (cRGD) peptides. However, the molecular details governing the interaction between cRGD-functionalized GO dots and integrins remain poorly understood. In this work, all-atom molecular dynamics simulations are employed to investigate the interaction between integrin α_V_β_3_ and a nanocarrier composed of a GO dot coated with polyethylene glycol (PEG) and functionalized with cRGD ligands. Multiple 1 μs simulation replicas are used to characterize both specific ligand recognition and non-specific nanocarrier/receptor interactions. The simulations show that cRGD binding within the integrin-binding pocket is stable, indicating that the nanocarrier does not impair receptor recognition. Beyond cRGD-mediated binding, both PEG-cRGD chains and GO itself establish additional contacts with the protein, whose nature and distribution are modulated by the relative orientation of the GO plane. Overall, the structural dynamics of integrin α_V_β_3_ remains preserved upon nanocarrier binding. These findings provide atomistic insights into the interplay between ligand-mediated and multivalent surface-mediated interactions of GO-based nanocarriers with integrins for the rational design of selective nanocarriers for cancer therapy.

## 1. Introduction

The limited selectivity of conventional anticancer therapies remains a major challenge in oncology [1]. Nanomedicine offers promising strategies to enhance therapeutic efficacy. Indeed, nanomaterials can be used to carry chemotherapeutic drugs and deliver them selectively, increasing drug concentration at cancer sites and improving cellular uptake through active targeting mechanisms. In this context, surface functionalization of nanomaterials with ligands that recognize receptors overexpressed on cancer cells has emerged as an effective approach to increase specificity and reduce off-target effects [2,3,4].

Among the most exploited cellular targets, integrin α_V_β_3_ plays a central role in cancer progression, angiogenesis, and metastasis [5,6,7,8,9]. This heterodimeric transmembrane protein receptor mediates cell-extracellular matrix interactions and is markedly overexpressed in several solid tumors while exhibiting low expression in healthy tissues [10]. The ligand-binding pocket of α_V_β_3_ recognizes the Arg-Gly-Asp (RGD) motif, a sequence present in various extracellular matrix proteins. Small cyclic RGD peptides have therefore been widely employed as targeting ligands to be conjugated on the surface of nanocarriers, owing to their high affinity to integrin α_V_β_3_, structural rigidity, and resistance to enzymatic degradation [11,12].

Graphene oxide (GO), a two-dimensional oxidized derivative of graphene, has attracted considerable attention as a nanoplatform for biomedical applications. Owing to its large surface-to-volume ratio, mechanical stability, aqueous dispersibility, and tunable surface chemistry, GO is an appealing candidate for targeted drug delivery [13,14,15] and photodynamic or photothermal cancer therapy [16,17,18,19]. Its surface contains both graphitic domains and oxidized regions decorated with oxygen-containing functional groups, enabling π–π stacking, hydrogen bonding, and electrostatic interactions with biomolecules. Furthermore, GO can be coated with polymers such as polyethylene glycol (PEG) to enhance biocompatibility and circulation time [15,20,21], and functionalized with targeting ligands, including cyclic RGD peptides, to achieve receptor-specific interactions [22,23,24,25,26,27,28]. Experimental studies on cRGD-functionalized GO nanomaterials have demonstrated enhanced integrin-mediated cellular uptake and targeting capability, supporting the use of cRGD as an effective strategy to confer receptor specificity to GO-based nanocarriers [29].

Despite the growing interest in GO-based nanocarriers functionalized with RGD ligands for targeting integrin-α_V_β_3_-positive tumors, an atomistic understanding of the interaction between GO and integrin receptors remains limited, as such details are challenging to detect with currently available experimental techniques. Some computational studies have investigated drug adsorption on GO surfaces [30,31,32], others have characterized RGD binding to integrin α_V_β_3_ [33,34] or the interaction of cRGD-conjugated inorganic nanoparticles with integrin α_V_β_3_ [35], and a recent study has characterized the membrane-mediated ligand-independent activation mechanism of integrin α_V_β_8_ by PEGylated GO [36]. However, the ligand-mediated interaction between PEGylated GO and integrins has not been explored yet at an atomistic resolution. In particular, it is unclear whether GO can directly interact with the integrin surface beyond the canonical RGD binding pocket, potentially influencing receptor conformation, accessibility, or engagement in integrin clustering.

In this work, we employ all-atom molecular dynamics (MD) simulations to investigate the interaction between a realistic GO-based nanocarrier and the extracellular domain of integrin α_V_β_3_—hereafter simply referred to as “integrin”. The nanocarrier is composed of a 5-nm wide GO dot, coated with PEG chains (MW = 1000 Da), which are in turn conjugated with cyclic c(RGDyK) ligands, abbreviated as “cRGD”. This nanocarrier design has already been used in a previous computational study by some of us [30] because it reproduces common experimental designs, where GO is usually coated with PEG chains for prolonged circulation time [21,22,37,38,39] and provided with cRGD targeting ligands [22]. Through multiple 1 µs-long replicas, we analyze both specific recognition at the ligand-binding pocket and non-specific interactions across the protein surface, assessing the role of the PEG chains and the other cRGD molecules in the interaction, as well as the effect of the orientation of the GO plane with respect to the protein surface. By providing atomistic insights into the mechanisms governing this nanocarrier/receptor interaction, the present study aims to contribute to the rational design of GO-based nanocarriers for targeted cancer therapy.

The description of the computational models and all computational details are provided in Section 2, while the results and discussion are presented in Section 3, followed by the conclusions in Section 4.

## 2. Methods

First, we discuss how the nanocarrier was modeled (Section 2.1). Second, we present the rigid receptor docking approach we adopted to dock one cRGD ligand into the binding pocket of integrin α_V_β_3_ (Section 2.2). Third, we list the details of the MD simulation (Section 2.3). Lastly, we present the methods used to analyze the MD simulations (Section 2.4). The systems were visualized using VMD (version 1.9.4a51) [40], and the plots were generated using Python (version 3.6.8).

### 2.1. Modeling of cRGD-Conjugated PEGylated GO Dot and of Integrin α_V_β_3_

**Figure 1:**
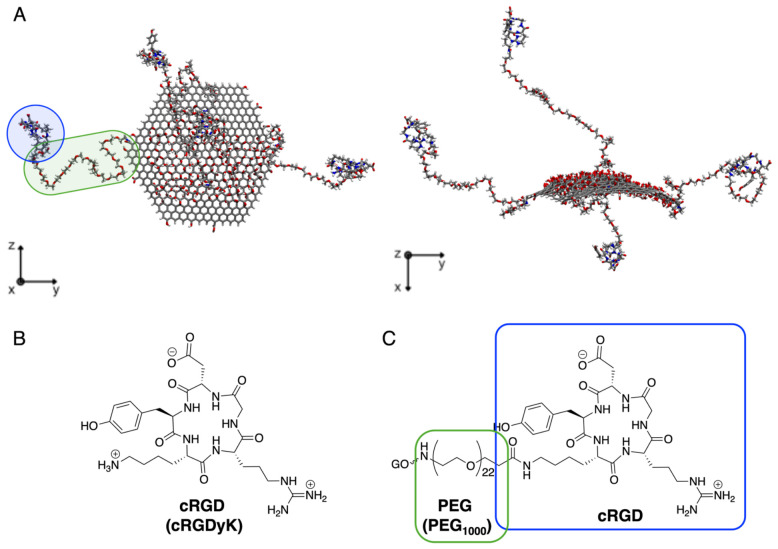
Initial structure—after minimization and before equilibration—of the GO-PEG-cRGD model used in this work: top view and side view (**A**), cRGD structural formula (**B**), and PEG-cRGD structural formula (**C**). Color code: C atoms in grey (licorice representation), O atoms in red (licorice representation), H atoms in white (licorice representation), and N atoms in blue (licorice representation).

The nanocarrier is a prototypic drug carrier composed of three main parts: (i) the GO dot, (ii) the PEG chains, and (iii) the cRGD ligands (Figure 1)—hereafter referred to as “GO-PEG-cRGD”. The three components are covalently attached, as represented in Figure 1.

To model GO, we used a 5 nm-wide model with a flake shape, with various oxygen-based groups on the surface, including hydroxyl and epoxy groups on the plane and carboxyl and phenolic groups at the edges, yielding a minimal formula of C_2.5_O_1_H_0.8_ which is typical for GO [41]. The functional groups are distributed into oxidized islands, leaving parts of the GO dot unoxidized, as they would be in pristine graphene, according to experimental evidence from microscopy studies [41]. The *MakeGraphitics* [42] Python library (v0.1.1) was used, allowing the creation of an *island* model of the GO dot, where the oxygen-based chemical groups are arranged into graphitic and oxidized islands. This Python library incorporates a random forest algorithm trained on ab initio calculation data [43]. Using this approach, a realistic GO dot structure was generated, whose chemical details can be found in Table S1 [30]. Notably, (i) we used a diameter of 5 nm, as this size is close to the smallest experimentally achievable dimension while still containing graphitic domains of realistic size (1–2 nm) [15,41,44], (ii) we set the C/O ratio to 2.5, because it reproduces the characteristic oxygen content of GO produced by the modified Hummers’ method for biomedical applications [15,21,37,45], and (iii) we tuned the ratio between edge and basal plane groups to agree with experimental values [46]. The same GO dot model was used in previous work by some of us [30].

Subsequently, four cRGD-conjugated PEG chains were covalently bonded on the GO dot model in agreement with experimentally determined polymer content (weight percentage of 25% wt) and the common practice of conjugating each polymer chain with a targeting ligand [22], as described in previous work by some of us [30]. Each PEG chain is 22 units long, or, equivalently, referred to as the PEG1000 chain, where the number in subscript denotes the approximate molecular weight (Da) of the PEG chain and is covalently conjugated to cRGD (Figure 1). Two chains were bonded to the surface of GO (epoxide ring opening) and two to the edges (amidation of carboxyl groups), resulting in a net charge of −4 *e* after assigning the protonation states corresponding to neutral pH. Specifically, all COO^−^ groups of GO and cRGD were deprotonated and the guanidinium groups of cRGD were protonated (Figure 1C). The resulting model corresponds exactly to the GO_i_-PEG-cRGD model presented in previous work by some of us [30], where we characterized its behavior in solution and its affinity with a chemotherapeutic drug (doxorubicin).

The structure of the integrin α_V_β_3_ extracellular segment was taken from the Protein Data Bank (code 4MMX) [47]. The co-crystallized ligand (fibronectin) was removed while the two co-crystallized water molecules in the ligand-binding pocket were kept, and the eight co-crystallized cations were modeled as Ca^2+^ ions, according to a common practice in integrin α_V_β_3_ modeling [34,48,49]. The protein structure was prepared using the *Protein Preparation Wizard* in Maestro (release 2021-1) [50], including the addition of hydrogen atoms and the modeling of missing residues. One cRGD ligand was docked in the binding pocket using the docking procedure described in the next section.

In this work, covalently attached groups are indicated with a dash (e.g., *GO-PEG-cRGD*), while non-covalently bound groups are represented with a slash (e.g., *GO-PEG-cRGD*/*integrin*). We indicate the cRGD at the integrin ligand-binding pocket as “pocket-bound cRGD” and any other cRGD conjugated to the nanodevice but not specifically bound to the integrin ligand-binding pocket as “non-pocket-bound cRGD”.

### 2.2. Docking of cRGD in the Ligand-Binding Pocket of Integrin α_V_β_3_

The docking calculations were performed in Maestro (release 2021-1). The cRGD ligand was prepared using the *LigPrep* tool and the grid generated using the *Receptor Grid Generation* tool. The rigid-receptor docking calculations were carried out at extra precision (XP) using the *Glide* software (release 2021-1) [51,52,53]. We obtained 6 docking poses for the cRGD into the binding pocket of integrin α_V_β_3_, which were very close in terms of docking score value (Table S2). Therefore, to identify a representative pose, we used two quantitative criteria taking as reference the RGD sequence of cilengitide, a cyclic peptide that was co-crystallized with the integrin α_V_β_3_ (PDB:1L5G) [54].

The first criterion is based on the three geometrical criteria proposed by Kostidis et al. [55] and by Othman et al. [56] to assess the biological activity of RGD-based ligands. These three criteria are named as *d*_1_, *d_2_*, and *θ* and represented in Figure S1. *d*_1_ is the distance between C_ζ_ and C_γ_ of Arg and Asp, respectively. *d*_2_ is the distance between the C_β_ of both Arg and Asp. *θ* is the angle between C_β_, C_α_ and C_β_ of Arg, Gly and Asp, respectively. To quantify the deviation in the values of the three geometrical criteria for the docking poses with respect to the values measured for the co-crystallized cilengitide, the following normalized Euclidean distance, named *Score*, was defined:

(1) $$Score\;=\;\sqrt{{\left(\frac{d_{1}-d_{1,0}}{d_{1,0}}\right)}^{2}+{\left(\frac{d_{2}-d_{2,0}}{d_{2,0}}\right)}^{2}+{\left(\frac{\theta -\theta_{0}}{\theta_{0}}\right)}^{2}}$$

where *d*_1,0_, *d*_2,0_, and *θ*_0_ are the values for the co-crystallized cilengitide.

The second criterion evaluates the structural similarity of the RGD motif in each docking pose against the cilengitide crystal structure, using the root mean square deviation (RMSD) computed over the heavy atoms of this common RGD motif. The *Score* and the RMSD were normalized using the standard formula:

(2) $$X_{norm}=\frac{X-X_{min}}{X_{max}-X_{min}}$$

where *X_norm_* is the normalized value of the original value *X*, while *X_min_* and *X_max_* are, respectively, the minimum and maximum values in the dataset. *X* represents either the RMSD or *Score* calculated for each docking pose and normalized to a range between 0 and 1. Based on normalized RMSD and *Score* in Figure S1, pose 6 was selected and used as the starting point structure in MM MD simulations.

### 2.3. Molecular Dynamics Simulation Details

To fully investigate the interactions of the nanocarrier with the extracellular domain of integrin α_V_β_3_, we performed a wide set of MD simulations. We carried out 3 MD replicas of the integrin in complex with the GO-PEG-cRGD nanocarrier (*GO-PEG-cRGD*/*integrin MD*). This system was compared against three reference MD simulations including (i) the isolated integrin α_V_β_3_ (*integrin MD*), (ii) the isolated GO-PEG-cRGD nanocarrier in aqueous solution, in the absence of the protein (*GO-PEG-cRGD MD*), or (iii) the integrin/cRGD complex (*cRGD*/*integrin MD*). MD simulations (i) and (ii) were used to characterize the intrinsic dynamics of the isolated receptor and nanocarrier, respectively; and the contribution of the pocket-bound cRGD ligand alone was assessed through the analysis of simulation (iii). A summary of all the performed simulations is reported in Table S3.

The initial structures for each simulation were prepared as follows: the GO-PEG-cRGD nanocarrier was built with the procedure in Section 2.1; the integrin α_V_β_3_ structure was prepared as described above in Section 2.1 before the docking calculations; the cRGD/integrin complex was obtained by docking as detailed in Section 2.2; the GO-PEG-cRGD/integrin complexes were constructed from the cRGD/integrin complex by conjugating the pocket-bound cRGD ligand to the GO-PEG-cRGD nanocarrier and by varying the initial orientation of the GO plane relative to the protein across the three replicas. In particular, the pocket-bound cRGD was conjugated to GO-PEG-cRGD by converting the amino group of cRGD lysine into an amide group connecting the ligand to one of the four PEG chains of the GO-PEG-cRGD nanocarrier (Figure 1C).

Every system was parametrized with CHARMM36 (and CGenFF) force field (FF) [57,58,59]. In particular, the integrin and the cRGD/integrin complex were prepared with the *Ligand Reader & Modeler* CHARMM-GUI module [60]. CHARMM36 FF [61,62,63] was used for the integrin and CGenFF [57,58,59] for the cRGD ligand, removing NBFIX correction for Ca^2+^ cations, according to previous work by some of us [35]. The nanocarrier was modeled with CGenFF parameters, which were assigned to PEG and cRGD ligands with the CHARMM-GUI *Ligand Reader & Modeler* [60] module and to GO with an in-house python script, according to CGenFF parameters used by Williams et al. [64]. CGenFF has been already used for modeling GO structures with the same type of functional groups [31,32,64].

Each system was solvated in an aqueous solution of NaCl (0.15 M), using the rigid CHARMM-modified TIP3P water model [63,65] in a cubic box with a side length of 120 Å for *GO-PEG-cRGD MD* and of 165 Å for all the other systems. A minimization of 5000 steps using the steepest descent algorithm was carried out, followed by a 2 ns NVT equilibration and an NPT production run. The temperature was set at 303.15 K by V-Rescale thermostat [66], with a coupling constant of 1.0 ps. The pressure was kept constant at 1 bar by a Parrinello-Rahman barostat [67] with a coupling constant of 5.0 ps. The Particle Mesh Ewald (PME) solver [68] handled the electrostatic interactions with a real-space cutoff of 12.0 Å, while van der Waals interactions were smoothly switched to zero using a force-switch function between an inner cutoff of 10 Å and an outer cutoff of 12 Å. Newton’s equations of motion were integrated with the leap-frog algorithm [69]. All covalent bonds involving a hydrogen atom were constrained using the LINCS algorithm [70,71]. Production runs were carried out for 100 ns for the *GO-PEG-cRGD MD*, for 500 ns for both the isolated *integrin MD* and the *cRGD*/*integrin MD*, and for 1000 ns for each of the three independent *GO-PEG-cRGD*/*integrin MD* replicas. The latter therefore account for a cumulative simulation time of 3 μs. All minimization, equilibration and production runs were performed using the GROMACS software package (version 2022.3), exploiting GPU acceleration [71].

As mentioned above, the three independent 1000 ns-long replicas—hereafter indicated with #1, #2, and #3—differ in the relative orientation between the integrin and the GO-PEG-cRGD nanocarrier. The different starting-point geometries were obtained by rotating and translating the GO plane with respect to the protein surface in VMD and solvating the structure afterwards, which may result in slight differences in the number of water molecules (Table S3).

### 2.4. Molecular Dynamics Simulation Analysis

The number of hydrogen bonds (H-bonds) was computed using GROMACS tools (version 2023.2) with a donor–acceptor cutoff distance of 3.5 Å and an acceptor–donor–hydrogen cutoff angle of 30°. Distances and the number of contacts were also calculated with GROMACS tools. The contact matrix was obtained with an in-house python script using the MDAnalysis library (version 2.0.0) [72,73]. The residue contact occupancy was calculated as the fraction of analyzed simulation frames in which a residue formed at least one contact with the counterpart, expressed as a percentage. Specifically, the occupancy was computed as N_contact_/N_frames_ × 100, where N_contact_ is the number of frames containing at least one heavy-atom contact between the residue and the counterpart (within 4 Å), and N_frames_ is the total number of analyzed frames. To quantitatively evaluate the flexibility of the protein residues, the Root Mean Square Fluctuation (RMSF) was computed with GROMACS tools after least-squares superimposition considering only Cα. All relevant intermolecular interactions were computed with GROMACS by re-running the MD simulations. The solvent accessible surface area (SASA) [74] was calculated with GROMACS tools, with a probe radius of 1.4 Å and a Van der Waals radius taken from Ref. [75] The SASA represents the area that is accessible to a solvent molecule, computed by rolling a spherical probe (representing a solvent molecule) over the molecule’s surface.

Trajectory frames were saved every 200 ps for subsequent analysis. For *GO-PEG-cRGD MD*, properties were averaged over the last 50 ns of the 100 ns simulation, whereas for *integrin MD* and *cRGD*/*integrin MD* averages were computed over the last 100 ns of the 500 ns simulation. For the *GO-PEG-cRGD*/*integrin MD*, analyses were performed over the last 500 ns of the 1000 ns runs.

## 3. Results and Discussion

To investigate the interaction between GO-PEG-cRGD (the nanocarrier) and the integrin (the receptor), we first prepared the model of the GO-based nanocarrier as a 5-nm GO dot with a flake shape, PEG coating and cRGD conjugation (GO-PEG-cRGD), aiming to reproduce the experimental data as close as possible [22]. The procedure followed to build the model and its structural features are described in Section 2.1. Secondly, we prepared the integrin structure and docked one cRGD ligand in the binding pocket, as detailed in Section 2.1 and Section 2.2. Finally, MD simulations were performed (see Table S3 for a complete list).

In the discussion of results, we primarily focus on *GO-PEG-cRGD*/*integrin MD* simulations, as these provide the most complete description of the interaction between the targeted nanocarrier and the receptor. The simulations of the isolated *integrin*, the *cRGD*/*integrin* complex, and the *GO-PEG-cRGD* nanocarrier serve as reference systems for highlighting the effects that arise from ligand conjugation and/or from the presence of the nanocarrier.

For *GO-PEG-cRGD*/*integrin MD* simulations, three initial geometries were generated, differing only in the relative orientation of the nanocarrier with respect to the receptor surface, and each was used for an independent 1 µs MD simulation—hereafter referred to as replicas. The three replicas were performed using distinct, physically plausible initial orientations of the GO flake relative to the protein. These configurations were designed to explore the effect of the initial nanocarrier orientation and should not be interpreted as statistical replicates of the same initial configuration or as an exhaustive sampling of the configurational ensemble.

**Figure 2:**
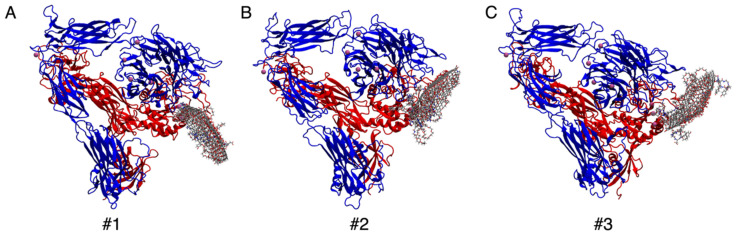
Configurations of the GO-PEG-cRGD/integrin complexes after 1 µs of MD simulation for *GO-PEG-cRGD*/*integrin MD* replicas (**A**) #1, (**B**) #2, and (**C**) #3. Color code: α_V_ in blue (cartoon representation), β_3_ in red (cartoon representation), C atoms in grey (licorice representation), O atoms in red (licorice representation), H atoms in white (licorice representation), N atoms in blue (licorice representation), Ca^2+^ ions in pink (van der Waals representation). Water and ions are not shown for clarity.

As it is clear from simple visual inspection of trajectories and from the final snapshots in Figure 2, the nanocarrier remained bound to the integrin throughout the simulations and did not detach within the 1 µs timescale. In fact, each initial configuration evolved toward a distinct and stable relative orientation that persisted until the end of the simulation. In replica #1, the GO plane is oriented perpendicular to the local receptor surface, whereas in replicas #2 and #3 it is oriented parallel to the surface with the convex and concave GO sides facing the protein, respectively. We emphasize that not only the nanocarrier remained in contact with the receptor surface throughout the simulations, but, more importantly, the pocket-bound cRGD was stably bound within the integrin ligand-binding pocket in all replicas for the entire simulation time.

**Figure 3:**
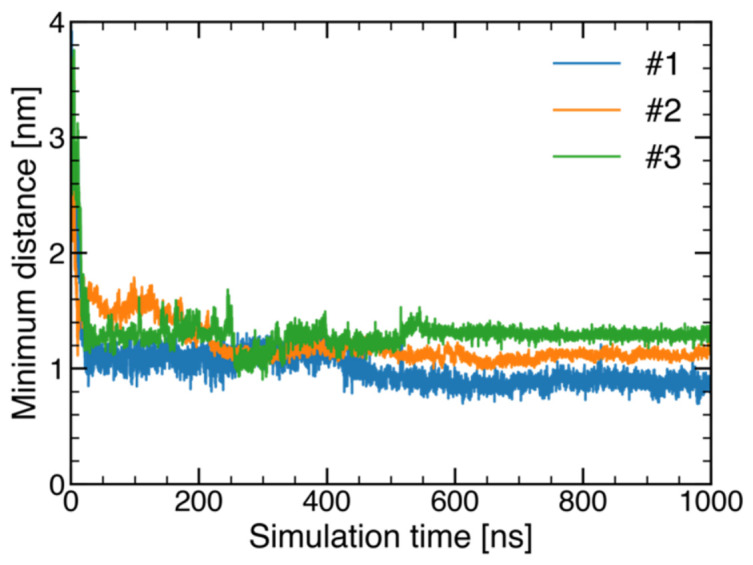
Minimum distance between the GO center of mass and the Ca^2+^ at MIDAS site of the integrin-binding pocket across the 1 µs *GO-PEG-cRGD*/*integrin MD* replicas #1, #2, and #3.

First, we monitored the distance between the GO center of mass and the central atom of the integrin-binding pocket, namely the Ca^2+^ ion located at the MIDAS site (see Figure 3). We observe that the nanocarrier rapidly approached the integrin-binding pocket during the early stages of each MD simulation and then fluctuated around a stable equilibrium distance. Based on this descriptor, the system was considered equilibrated after approximately 500 ns; therefore, all time-averaged quantities were calculated over the 500 ns–1 µs interval.

In the following, the overall GO-PEG-cRGD/integrin interaction is analyzed by distinguishing interactions occurring at the ligand-binding pocket of integrin, located at the interface between subunits α and β (Section 3.1), from those involving the remaining protein surface (Section 3.2).

### 3.1. Specific Interaction at the Ligand-Binding Pocket

Regarding the specific interactions between the pocket-bound cRGD and the integrin at the ligand-binding pocket, the same interaction pattern previously reported in computational [35] and experimental studies [54] is observed. In particular, on one side of the binding pocket, the cRGD aspartate side chain adopts a bidentate binding mode, in which one carboxylate oxygen coordinates the Ca^2+^_MIDAS_, while the second oxygen forms a H-bond with nearby amino acid residues. This corresponds to the *bridging* binding mode [35] previously identified for RGD-based ligands co-crystallized with the integrin α_V_β_3_ [54] (Figure S2). On the opposite side of the pocket, the cRGD arginine side chain forms multiple H-bonds with aspartate residues of the protein.

Overall, the average number of H-bonds between the pocket-bound cRGD and the integrin ranges from 2.7 to 4.6 across the different replicas, including the number computed during *cRGD*/*integrin MD* (Table 1). These interactions include the characteristic aspartate- and arginine-mediated H-bonds, as well as occasional contributions from the other two residues forming the cRGD cycle, lysine and D-tyrosine. Since no statistically significant difference in the number of H-bonds is observed among the three *GO-PEG-cRGD*/*integrin MD* replicas and in comparison with *cRGD*/*integrin MD*, the simulations indicate that neither the presence of the nanocarrier nor its orientation relative to the receptor affects cRGD recognition within the integrin-binding pocket.

Consistent with the structural analyses, the total cRGD/integrin interaction energies, including van der Waals and electrostatic contributions, remain within the same range, considering the associated uncertainties, across all GO-PEG-cRGD/integrin replicas and the cRGD/integrin reference system (Table S4). This indicates that nanocarrier conjugation does not substantially alter the energetic stabilization of cRGD within the integrin-binding pocket. These values should be considered as qualitative indicators of ligand/receptor interaction preservation rather than quantitative estimates of binding affinity.

Overall, our results show that the ligand remains stably anchored within the integrin-binding pocket even in the presence of the nanocarrier, regardless of the orientation of the GO plane relative to the protein surface. However, different relative orientations—likely arising from distinct approach orientations of GO toward the integrin—can lead to differences in the overall interaction between the nanocarrier and the protein. This effect is analyzed in the next section.

### 3.2. Non-Specific Interactions Between the Nanocarrier and the Receptor Surface

Having established that nanocarrier conjugation does not compromise cRGD specific recognition, the next question concerns the extent to which the GO-PEG-cRGD nanocarrier interacts with regions of the receptor outside the ligand-binding site. While experimental studies shown that GO functionalized with integrin-targeting ligands is internalized more efficiently by integrin-expressing cancer cells than by those lacking these receptors [26,76], they do not provide atomistic information on the interactions formed between the nanocarrier and the protein surface. The present MD simulations offer a detailed characterization of these additional contacts.

The number of H-bonds formed with the integrin surface (Table 1) indicates that both the cRGD ligands and the PEG chains contribute to the interaction when the GO plane is oriented parallel to the receptor surface. Even the GO sheet itself participates in H-bond interactions with the receptor in replica #2. In contrast, these contributions are minimal or absent in replica #1. This difference can be rationalized by simple geometric considerations. In replica #1 and #3, the number of H-bonds between pocket-bound cRGD and integrin matches that of cRGDs/integrin pair because only the pocket-bound cRGD interacts with the protein. The non-pocket-bound cRGDs are instead solvated or interacting with the GO surface (Figure S3).

Since GO is a heterogeneous material, whose synthesis can be tuned to achieve different degrees of oxidation, it is particularly important to identify which oxygen-containing functional groups are primarily responsible for H-bond formation with the integrin surface. In all three replicas, independently of the orientation of the GO plane relative to the protein surface, approximately 70% of the GO/integrin H-bonds reported in Table 1 are formed by hydroxyl groups, representing a much larger contribution compared with epoxide and carboxyl groups.

**Figure 4:**
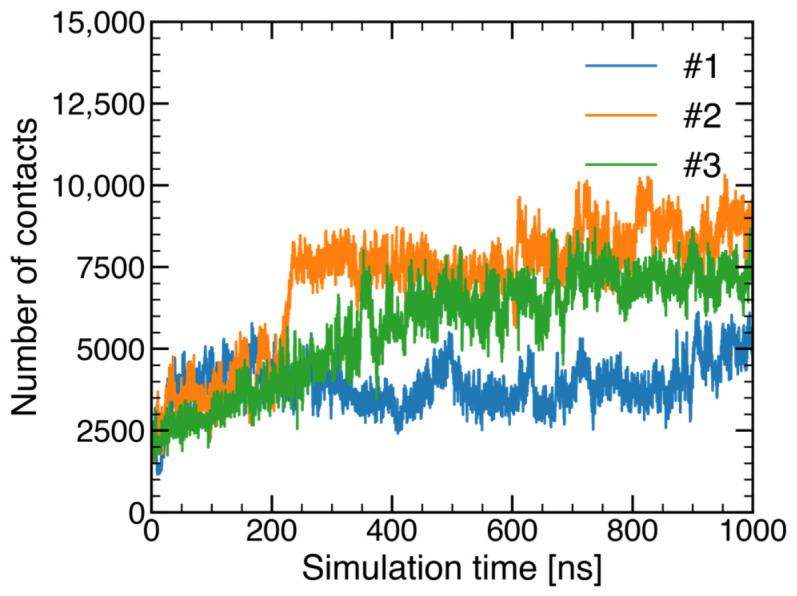
Number of contacts between GO-PEG-cRGD and integrin along the 3 *GO-PEG-cRGD*/*integrin MD* replicas, calculated within a cutoff of 4 Å. Each contact is counted as one, without any approximation or simplification.

To characterize the overall interaction, we quantified the number of contacts between the nanocarrier and the protein (Figure 4), which are based solely on a distance cutoff (4 Å) without any directionality constraint, unlike H-bonds. As expected, the number of contacts increases over the course of the simulation and slightly differs across the replicas, i.e., depending on the orientation of the GO-PEG-cRGD nanocarrier.

During the production phase (after 500 ns), the average number of contacts is 4000 ± 700 for replica #1, 8200 ± 800 for replica #2, and 6900 ± 700 for replica #3. The effect of the GO plane orientation is evident: the number of contacts is lower when the GO plane is oriented perpendicular to the protein surface (replica #1) and higher when it is parallel (replicas #2 and #3), because of the greater GO surface area exposed to the receptor in this orientation.

It is interesting to note that, during the production phase in all our simulations, the nanocarrier interacts only with the β-propeller domain of chain α_V_ and the βA domain of chain β_3_, as shown by the contact matrices in Figure S4. The nanocarrier contacts largely overlapping ranges of protein residues across the different replicas, with the exception of replica #3, where additional interactions are detected with residues in the 50–100 range of the β-propeller domain, while contacts involving residues in the 300–350 range of the βA domain are reduced or absent.

GO always participates in the interaction in all replicas, together with the pocket-bound cRGD, as expected. The other cRGD molecules and PEG chains contribute to the interaction to a lesser extent.

Moreover, in Table S5 we classified the nature of the protein residues contacted by GO-PEG-cRGD. The majority of interacting residues are neutral, with a preference for polar over non-polar side chains, followed by negatively charged residues and, lastly, positively charged residues. Additionally, sugar molecules located at the integrin glycosylation sites, which were retained from the original X-ray crystal structure, contribute to the nanocarrier interaction in replicas #1 and #2.

From an energetic perspective, we confirm that the strongest interactions occur when the GO approaches the protein surface in a parallel orientation, according to the GO-PEG-cRGD/integrin non-bonded interaction energies in Table 2. Specifically, replicas #2 and #3 show stronger interaction energies between the nanocarrier and the protein. Among these, the interaction is strongest in replica #2, where the convex side of the GO surface faces the protein. Therefore, in this case a slight difference is observed also between the two sides with which GO can approach the receptor.

Given that the interaction between GO-PEG-cRGD and integrin extends well beyond the ligand-binding pocket where a single cRGD ligand is docked, we evaluated its effect both on the solvation of the system and on the flexibility of the protein.

Starting from the investigation of the effect of the nanocarrier/protein interaction on the solvation of each component, the analysis is based on the energies reported in Table 2 and on the solvent-accessible surface area (SASA) evaluation in Table 3. We observe only minor variations in the solvation across the different configurations. In particular, the nanocarrier shows a slight tendency to be more solvated in replica #1, where it adopts a perpendicular orientation with respect to the protein surface. Despite this trend, the SASA remains comparable between the isolated components and the corresponding complexes (Table 3), indicating that the nanocarrier adsorption does not substantially alter the overall solvent exposure of the integrin. This is further supported by the consistent integrin/solvent non-bonded interaction energies across all replicas (Table 2).

**Figure 5:**
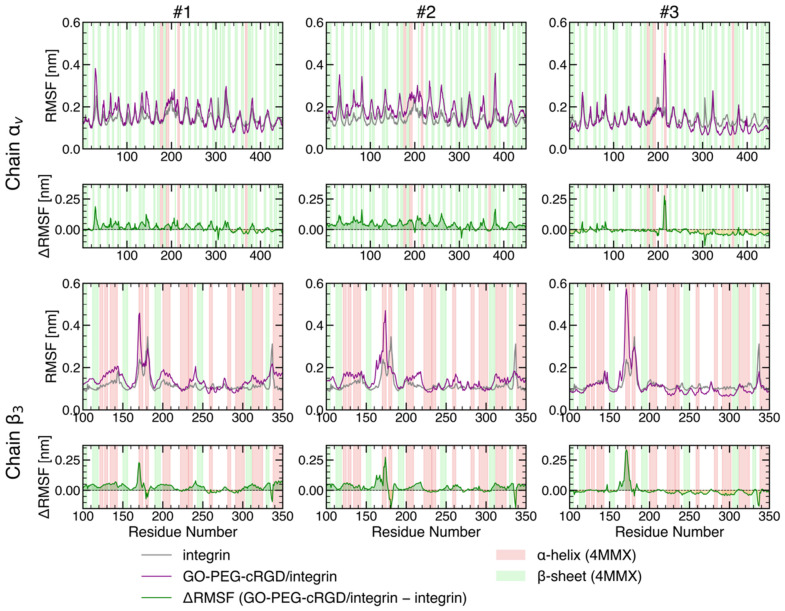
RMSF analysis for the β-propeller domain (residues 1–450 of chain α_V_) and the βA domain (residues 100–350 of chain β_3_). Each column corresponds to a different *GO-PEG-cRGD*/*integrin MD* replica. For each domain, both the absolute RMSF values for the isolated integrin (grey) and for the GO-PEG-cRGD/integrin complex (purple), averaged over the production phase, as well as the difference between the two systems (green), are reported. Vertical shaded regions indicate α-helices (red) and β-sheets (green). The ΔRMSF plot is colored in green where integrin residues are more mobile in the complex than in the isolated protein, and in yellow in the opposite case. The corresponding RMSF analysis can be found in Figure S6.

Lastly, we report the root-mean-square fluctuation (RMSF) of integrin residues in Figure 5 to assess how the presence of the nanocarrier affects the flexibility of protein residues, with particular focus on those in the integrin head domains forming the ligand-binding pocket, as these are the only domains involved in the interaction with the nanocarrier according to contact analysis above. We compared the differences in RMSF (ΔRMSF): (1) between the GO-PEG-cRGD/integrin complexes and isolated integrin; and (2) between the cRGD/integrin complex and isolated integrin (Figure 5 and Figure S6, respectively). Overall, the ΔRMSF profiles are qualitatively similar in the two cases, indicating that the nanocarrier does not introduce new regions of enhanced flexibility relative to cRGD binding alone. The main difference is a modest increase in fluctuation amplitude in the GO-PEG-cRGD systems, on the order of ~1–2 Å, across specific regions of the receptor.

Furthermore, no clear dependence on the initial nanocarrier orientation is observed, although replica #3 displays slightly lower ΔRMSF absolute values on average, when compared to the other orientations in replicas #1 and #2. This suggests a reduced perturbation of the protein dynamics in this configuration. Together, these observations suggest that this specific parallel orientation may lead to a slightly more compact and less dynamic nanocarrier/receptor interaction, resulting in minimal structural perturbation of the receptor.

## 4. Conclusions

In this work, atomistic MD simulations on the microsecond timescale were employed to investigate the interaction between a realistic nanocarrier model (PEGylated GO dot functionalized with cyclic RGD ligands) and its target receptor (extracellular domain of integrin α_V_β_3_). The simulations provide atomistic insights into the interplay between specific ligand-mediated recognition and non-specific nanocarrier/protein interactions.

The results show that the pocket-bound cRGD remains stably bound to the integrin-binding pocket regardless of the orientation adopted by the GO-PEG-cRGD nanocarrier when approaching the protein. The comparison with the cRGD/integrin reference system and with the experimentally characterized (X-ray) binding mode of cRGD ligands indicates that the conjugation of the ligand to the GO nanocarrier does not impair binding stability during the simulated timescale. This finding is in line with previous experimental studies on cRGD-functionalized GO nanomaterials, which have demonstrated that cRGD conjugation enables integrin-mediated recognition and cellular targeting, supporting the potential of PEGylated GO as a platform for RGD-targeted biomedical applications [29]. Further experimental and computational investigations will be required to determine whether GO conjugation affects quantitative binding properties of cRGD, such as affinity and kinetic parameters.

Beyond the specific ligand/receptor interaction, we find that the GO nanocarrier establishes additional transient contacts with regions of the protein surface outside the binding pocket. These interactions depend on the relative orientation of the GO plane with respect to the protein surface in the head region, with parallel configurations generally leading to more extensive contacts than perpendicular ones. Nevertheless, the overall interaction pattern remains qualitatively similar across the different orientations investigated, suggesting that the nanocarrier can adapt to the protein surface without compromising the targeted ligand recognition. Notably, the three simulated configurations provide representative limiting cases of the interaction landscape. Transitions between different GO plane orientations may occur on longer timescales, such as experimental timescales. Whether the configurations described in this study converge to a single preferred interaction mode or represent multiple metastable states of comparable stability remains an open question that would require significantly longer simulations long simulations or enhanced sampling methods.

Importantly, the presence of the nanocarrier induces only slight changes in the structural dynamics of integrin α_V_β_3_. The observed differences in residue fluctuations are small (typically on the order of 1–2 Å for selected residues), and no evidence of receptor destabilization or large conformational rearrangements is observed on the microsecond timescale. These findings indicate that GO acts primarily as a carrier for cRGD presentation, while PEGylation likely limits excessive non-specific adsorption to the protein surface and preserves selective receptor recognition. Notably, these conclusions were obtained using experimentally derived PEG and cRGD ligand amounts in our model [22]. Future studies could investigate additional design parameters, including PEG density, cRGD ligand density, and GO size, to further elucidate their impact on GO-based nanocarrier/integrin interactions and optimize targeting performance.

Overall, this study provides a molecular-level characterization of GO-PEG-cRGD interactions with integrin α_V_β_3_ and contributes to the rational design of graphene-based nanocarriers for targeted cancer therapy.

## Figures and Tables

**Table 1:** Number of H-bonds between GO-PEG-cRGD (total number, split by component) and integrin or water molecules. The average and standard deviation were calculated over the production phase of the *cRGD*/*integrin MD* (400–500 ns) and of the *GO-PEG-cRGD*/*integrin MD* replicas (500–1000 ns).

H-Bond Pair	cRGD/Integrin	#1	#2	#3
pocket-bound cRGD/integrin	3.0 ± 1.0	4.6 ± 1.0	2.7 ± 0.7	4.2 ± 1.1
cRGDs */integrin	-	4.6 ± 1.0	4.0 ± 1.3	4.3 ± 1.1
PEG/integrin	-	0.3 ± 0.6	1.7 ± 1.1	1.7 ± 1.0
GO/integrin	-	0.4 ± 0.6	4.2 ± 1.5	0.7 ± 0.7
GO-PEG-cRGD/integrin	-	5.3 ± 1.3	9.9 ± 2.0	6.7 ± 1.6
GO-PEG-cRGD/water	-	270 ± 10	260 ± 10	260 ± 10

* cRGDs are all cRGD molecules, including the pocket-bound cRGD and non-pocket-bound cRGDs.

**Table 2:** Non-bonded interaction energy between different components of the system divided into their electrostatic (Coulomb potential) and van der Waals (LJ potential) contributions. The average and standard deviation were calculated over the production phase of the *GO-PEG-cRGD*/*integrin MD* replicas (500–1000 ns). The solvent includes water and ions.

Non-Bonded Interaction Energy (kcal/mol)		#1	#2	#3
GO-PEG-cRGD/integrin	Electr.	−160 ± 10	−240 ± 30	−180 ± 20
	vdW	−60 ± 11	−130 ± 10	−100 ± 10
GO-PEG-cRGD/solvent	Electr.	−2260 ± 70	−2140 ± 70	−2050 ± 60
	vdW	−540 ± 30	−480 ± 30	−510 ± 20
Integrin/solvent	Electr.	−36,000 ± 400	−36,000 ± 400	−36,000 ± 300
	vdW	−2630 ± 90	−2600 ± 90	−2500 ± 90

**Table 3:** Solvent accessible surface area (SASA) for GO-PEG-cRGD and integrin, either isolated in solution (*integrin MD* or *GO-PEG-cRGD MD*) or in complex (*GO-PEG-cRGD*/*integrin MD*). The average and standard deviation were calculated over the production phase * of each MD simulation. The time evolution over the three replicas is shown in Figure S5.

SASA [nm^2^]	Isolated	#1	#2	#3
Integrin	774 ± 6	779 ± 7	800 ± 10	774 ± 8
GO-PEG-cRGD	95 ± 3	97 ± 4	94 ± 4	94 ± 2

* Refer to Section 2.4 for the definition of the production phase for each system.

## Data Availability

The original contributions presented in this study are included in the article and Supplementary Materials. Further inquiries can be directed to the corresponding authors.

## Supplementary Materials

The following supporting information can be downloaded at: https://www.mdpi.com/article/10.3390/nano16140896/s1. Figure S1: *Score* of the docking poses and criteria used to calculate *Score*; Figure S2: Distance between Ca^2+^_MIDAS_ and the two oxygen atoms of cRGD aspartate carboxylate group in *GO-PEG-cRGD/integrin MDs*; Figure S3: H-bonds in *GO-PEG-cRGD*/*integrin MDs*; Figure S4: Contact matrix in *GO-PEG-cRGD*/*integrin MDs*; Figure S5: SASA in *GO-PEG-cRGD*/*integrin MDs*; Figure S6: RMSF in *cRGD*/*integrin MD* compared to *integrin MD*. Table S1: Characteristics of the GO dot model; Table S2: *GlideScore* values; Table S3: Summary of MD simulations; Table S4: Non-bonded interaction energy in *cRGD*/*integrin MD* and in *GO-PEG-cRGD*/*integrin MDs*; Table S5: Nature of contacts in *GO-PEG-cRGD*/*integrin MDs*.

## Abbreviations

The following abbreviations are used in this manuscript:

MD	Molecular dynamics
cRGD	c(RGDyK)
GO	Graphene oxide
PEG	Polyethylene glycol
RMSF	Root Mean Square Fluctuation
SASA	Solvent accessible surface area
