# Supplementary material for: Atomistic Insights into Graphene Oxide Dot Interactions with Integrin αVβ3 from Microsecond Simulations
Source: Nanomaterials (Basel). 2026 Jul 22;16(14):896. doi: 10.3390/nano16140896 (PMC13415017; doi:10.3390/nano16140896)
Supplement: Supplementary file 1 [file nanomaterials-16-00896-s001.zip › nanomaterials-4444949-supplementary.pdf]

# Supplementary Materials

# Atomistic Insights into Graphene Oxide Dot Interactions with Integrin αvβ3 from Microsecond Simulations

**Giulia Frigerio [1,2,*], Jules Grollier [1], Paulo Siani [1,2], Edoardo Donadoni [1,2] and Cristiana Di Valentin [1,2,*]**

[1] Department of Materials Science, University of Milano-Bicocca, Via R. Cozzi 55, 20125 Milan, Italy; jules.grollier@uni-saarland.de (J.G.); paulo.siani@unimib.it (P.S.); edoardo.donadoni@unimib.it (E.D.)
[2] BioNanoMedicine Center NANOMIB, University of Milano-Bicocca, 20125 Milan, Italy
* Correspondence: giulia.frigerio@unimib.it (G.F.); cristiana.divalentin@unimib.it (C.D.V.)

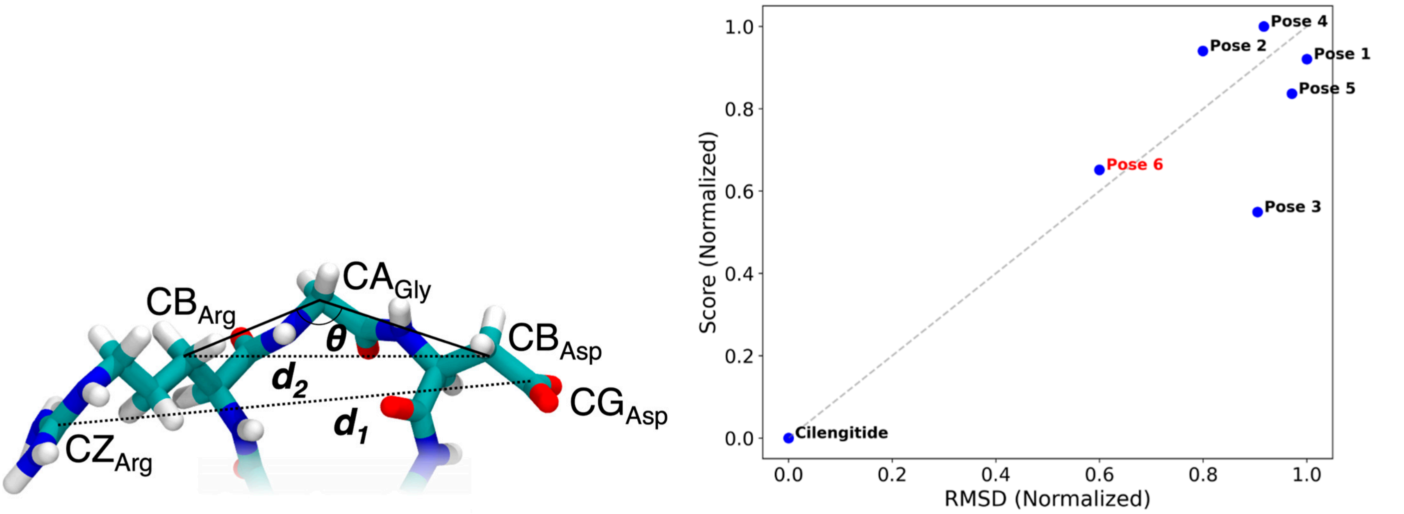


**Figure S1. (Right)** Representation of the three criteria used to calculate the *Score*. **(Left)** *Score* of the docking poses against their RMSD, where co-crystallized cilengitide is the reference structure.

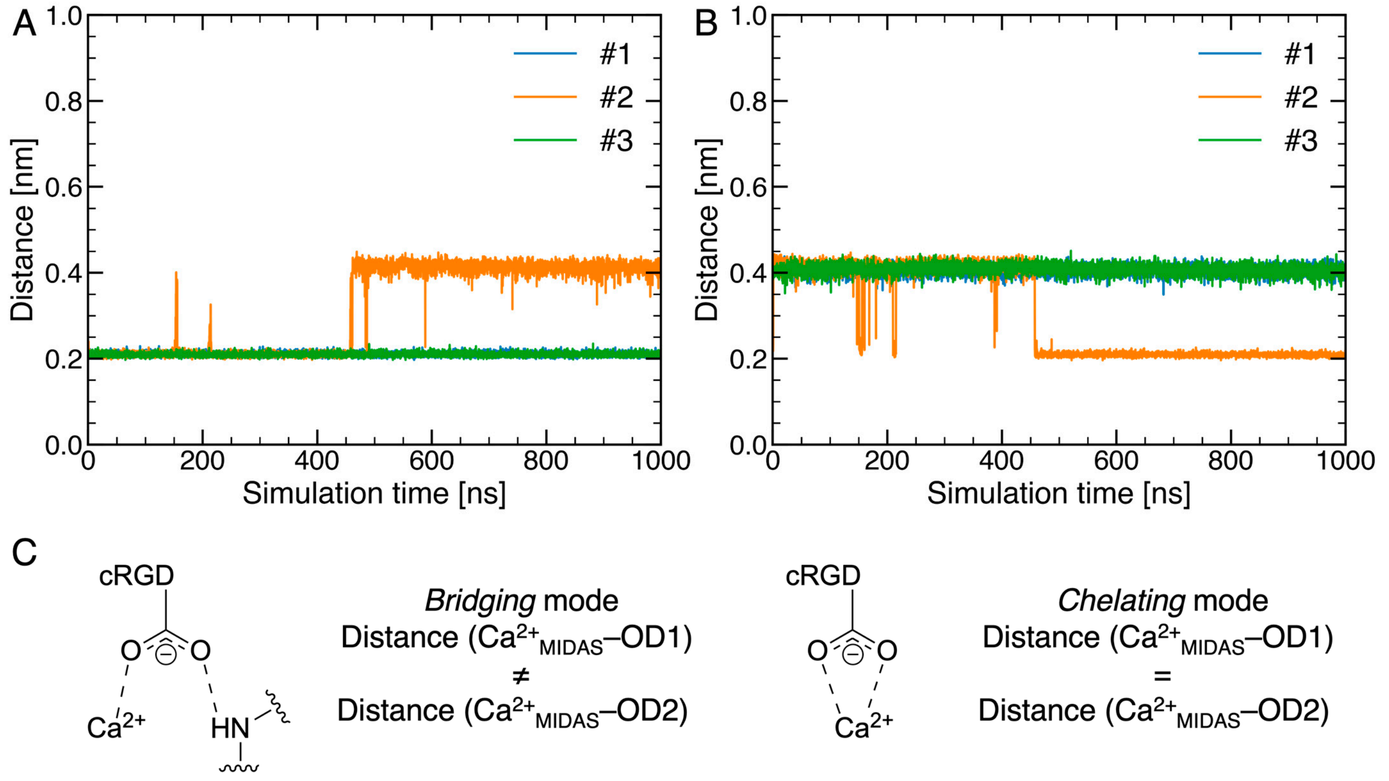


**Figure S2.** Time evolution of the distance between the $Ca^{2+}_{MIDAS}$ and the two oxygen atoms of cRGD aspartate carboxylate group, referred to as OD1 (**A**) and OD2 (**B**) along each *GO-PEG-cRGD/integrin MD* replica and scheme of different binding modes (**C**).

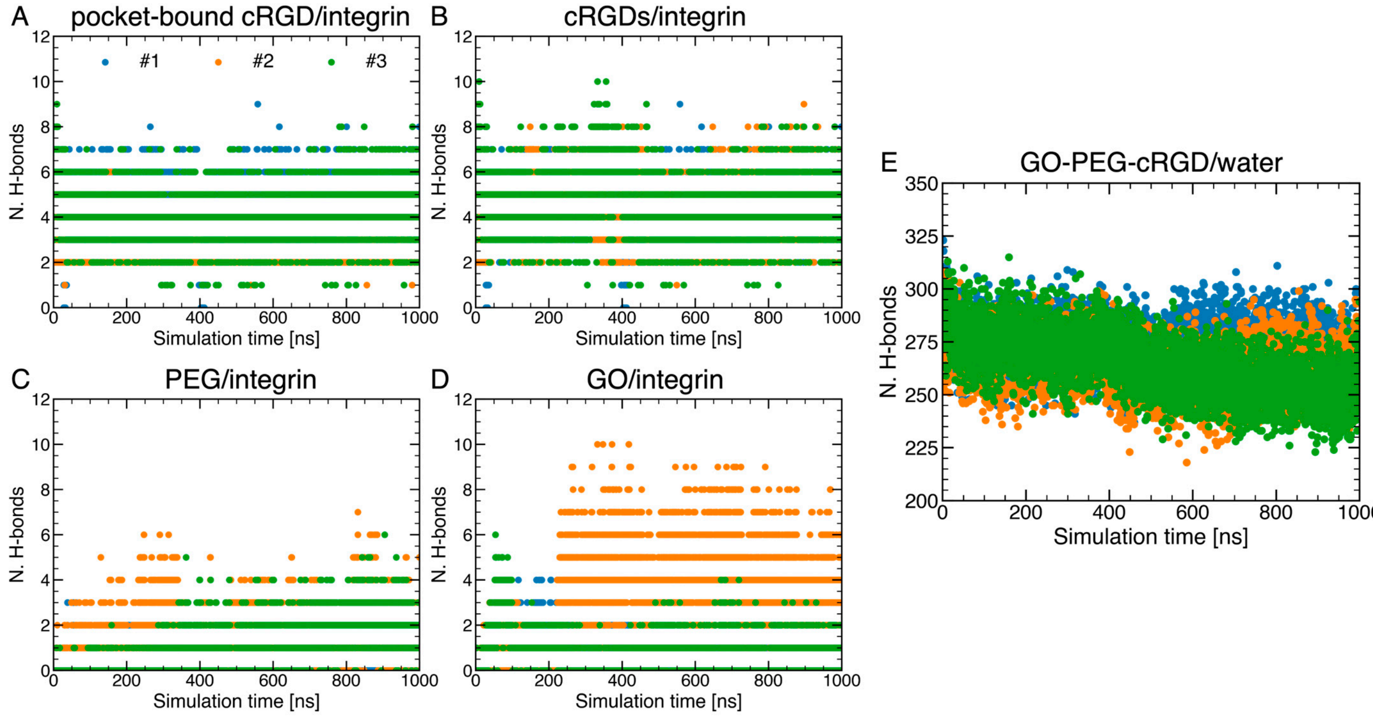


**Figure S3.** Time evolution of the number of H-bonds along each *GO-PEG-cRGD/integrin MD* replica for pocket-bound cRGD/integrin (**A**), cRGDs/integrin (**B**), PEG/integrin (**C**), GO/integrin (**D**) and GO-PEG-cRGD/water (**E**) pairs. In panel (**B**) cRGDs stands for all cRGD molecules, including the pocket-bound cRGD and non-pocket-bound cRGDs.

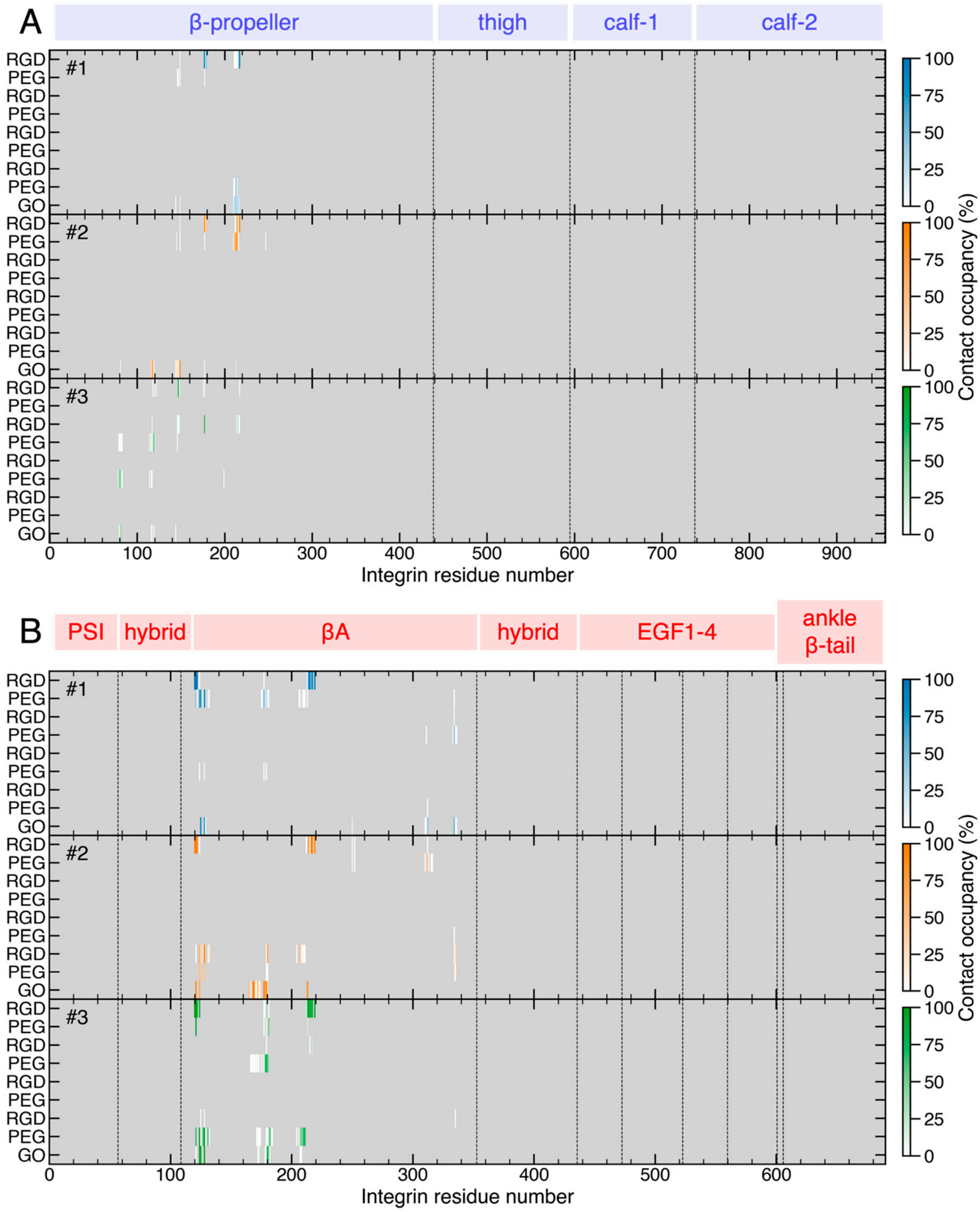


**Figure S4.** Contact matrix between GO-PEG-cRGD components and receptor residues for integrin chain αv (**A**) and chain β3 **(B)** colored by occupancy, averaged over the production phase (last 500 ns) for each *GO-PEG-cRGD/integrin MD* replica (4 Å cutoff). The first RGD starting from the top of each subfigure is the pocket-bound cRGD. A gray background indicates regions with no detected contacts.

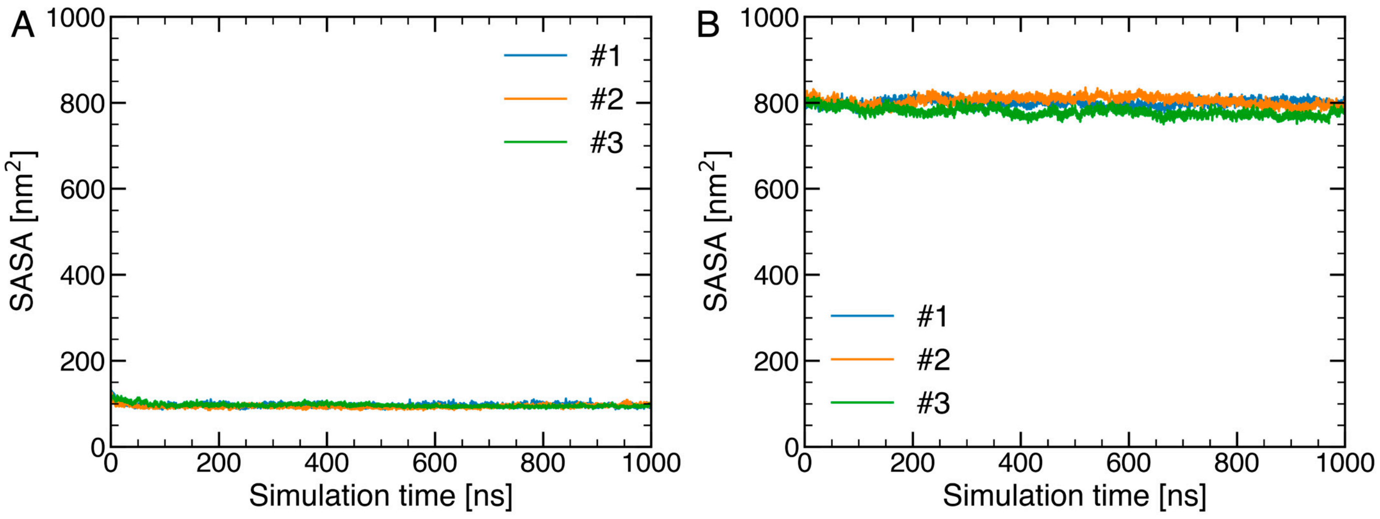


**Figure S5**. Time evolution of SASA of GO-PEG-cRGD (**A**) and integrin (**B**) along each *GO-PEG-cRGD/integrin MD* replica.

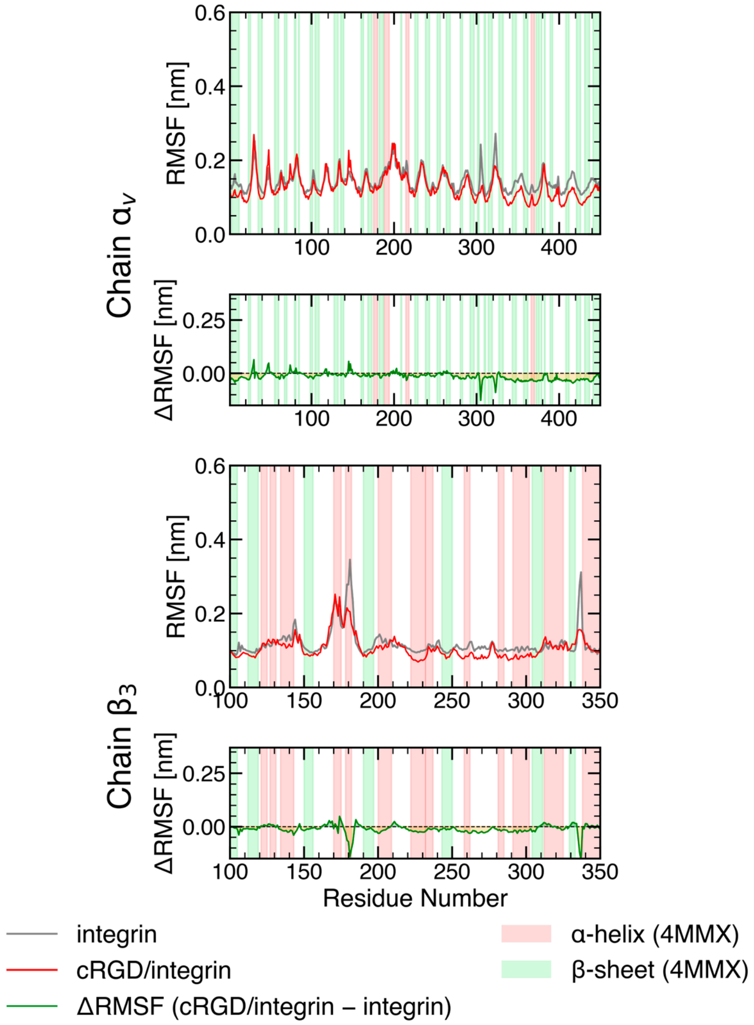


**Figure S6.** RMSF analysis for the β-propeller domain (residues 1-450 of chain $\alpha$v) and the βA domain (residues 100-350 of chain $\beta_3$) in *cRGD/integrin MD* with respect to in *integrin MD*. For each domain, both the absolute RMSF values for the isolated integrin and for the cRGD/integrin complex, averaged over the production phase, as well as the difference between the two systems, are reported. Vertical shaded regions indicate $\alpha$-helices and β-sheets. The ΔRMSF plot is colored in green where integrin residues are more mobile in the complex than in the isolated protein, and in yellow in the opposite case.

**Table S1.** Characteristics of GO dot model used in this work.

| **C number** | **607** |
|---|---|
| **O number** | 243 |
| **H number** | 187 |
| **Total atoms number** | 1037 |
| **C/O ratio** | 2.5 |
| **H/O ratio** | 0.80 |
| **mass [g mol$^{-1}$]** | 11367 |
| **$OH_{phenol}$** | 7 |
| **COOH** | 1 |
| **$COO^-$** | 6 |
| **$OH_{basal}$** | 133 |
| **Epoxy** | 89 |
| **Edge/plane ratio** | 0.06 |

**Table S2.** Docking score values, namely *GlideScore* from Maestro.

| **Pose** | ***GlideScore* [kcal/mol]** |
|---|---|
| 1 | –6.98 |
| 2 | –6.65 |
| 3 | –5.14 |
| 4 | –4.73 |
| 5 | –4.53 |
| 6 | –4.27 |

**Table S3.** Summary of the performed MD simulations used to study the effect of the binding of the nanocarrier to the integrin $\alpha v\beta_3$. $N_{atoms}$ is the total number of atoms in the MD simulation. $N_{waters}$ is the number of water molecules. $N_{ions}$ is the total number of buffer ions ($Cl^-$ and $Na^+$).

| **MD simulation** | **Production time [ns]** | **Box length [Å]** | **$N_{atoms}$** | **$N_{waters}$** | **$N_{ions}$** |
|---|---|---|---|---|---|
| *integrin* | 500 | 165 | 422,935 | 132,096 | 783 |
| *cRGD/integrin* | 500 | 165 | 423,022 | 132,096 | 784 |
| *GO-PEG-cRGD* | 100 | 120 | 169,181 | 55,614 | 316 |
| *GO-PEG-cRGD/integrin* (#1) | 1000 | 165 | 440,137 | 137,133 | 851 |
| *GO-PEG-cRGD/integrin* (#2) | 1000 | 165 | 440,143 | 137,135 | 851 |
| *GO-PEG-cRGD/integrin* (#3) | 1000 | 165 | 440,167 | 137,143 | 851 |

**Table S4.** Non-bonded interaction energy between pocket-bound cRGD and integrin or solution divided into their electrostatic (Coulomb potential) and van der Waals (LJ potential) contributions for *cRGD/integrin MD* and for the 3 replicas of *GO-PEG-cRGD/integrin MD* simulations. Average and standard deviation are calculated over the production phase of the MD simulations. The solvent includes water and ions.

| Non-bonded interaction energy (kcal/mol) | | cRGD/integrin | #1 | #2 | #3 |
|---|---|---|---|---|---|
| **pocket-bound cRGD/integrin** | Electr. | −160±10 | −146±8 | −140±8 | −130±10 |
| | vdW | −15±5 | −16±4 | −13±4 | −19±5 |
| **pocket-bound cRGD/solvent** | Electr. | −150±20 | −100±10 | −120±10 | −50±20 |
| | vdW | −16±6 | −9±5 | −15±5 | −13±4 |

**Table S5.** Chemical nature of integrin residues (including sugars at post-translational glycosylation sites) involved in contacts with GO-PEG-cRGD in the production phase (last 500 ns) for each GO-PEG-cRGD/integrin MD replica, based on per-residue contact occupancy analysis. The total number of residues with a non-zero contact occupancy is reported, along with the percentage of which belongs to positively charged amino acids (Arg, Lys, His), negatively charged amino acids (Asp, Glu), neutral polar amino acids (Ser, Thr, Asn, Gln, Tyr, Cys, Trp), neutral non-polar amino acids (Ala, Val, Leu, Ile, Met, Pro, Phe, Gly), and sugars.

| Replica | Total residues [n.] | Positive [%] | Negative [%] | Neutral polar [%] | Neutral non-polar [%] | Sugars [%] |
|---|---|---|---|---|---|---|
| #1 | 57 | 11 | 18 | 42 | 23 | 7 |
| #2 | 75 | 15 | 19 | 31 | 25 | 11 |
| #3 | 68 | 13 | 25 | 32 | 29 | 0 |
